# Vaccination differences among U.S. adults by their self-identified sexual orientation, National Health Interview Survey, 2013–2015

**DOI:** 10.1371/journal.pone.0213431

**Published:** 2019-03-07

**Authors:** Anup Srivastav, Alissa O’Halloran, Peng-Jun Lu, Walter W. Williams, Sonja S. Hutchins

**Affiliations:** 1 Leidos Inc., Atlanta, Georgia, United States of America; 2 Immunization Services Division, National Center for Immunization and Respiratory Diseases, Centers for Disease Control and Prevention, Atlanta, Georgia, United States of America; 3 Influenza Division, National Center for Immunization and Respiratory Diseases, Centers for Disease Control and Prevention, Atlanta, Georgia, United States of America; 4 Morehouse School of Medicine, Atlanta, Georgia, United States of America; University of Pennsylvania, UNITED STATES

## Abstract

**Introduction:**

Very few studies have explored the associations between self-identified sexual orientation and comprehensive vaccination coverage. Most of the previous studies that reported health disparities among lesbian, gay and bisexual populations were not based on a nationally representative sample of U.S. adults, limiting the generalizability of the findings. Starting in 2013, the National Health Interview Survey (NHIS) included questions to ascertain the adult’s self-identified sexual orientation that allowed national level vaccination estimation by sexual orientation. This study examined associations of self-reported vaccination coverage for selected vaccines among U.S. adults by their sexual orientation.

**Methods:**

We analyzed combined data from 2013–2015 NHIS, a nationally representative probability-based health survey of the noninstitutionalized U.S. population ≥18 years. For vaccines other than influenza, weighted proportions were calculated. Influenza coverage was calculated using the Kaplan-Meier procedure. Multivariable logistic regression models were used to calculate adjusted prevalence differences for each vaccine overall and stratified by sexual orientation and to identify factors independently associated with vaccination.

**Results:**

Significant differences were observed by sexual orientation for self-reported receipt of human papillomavirus (HPV), hepatitis A (HepA), hepatitis B (HepB), and influenza vaccination. Bisexual females (51.6%) had higher HPV coverage than heterosexual females (40.2%). Gay males (40.3% and 53.6%, respectively) had higher HepA and HepB coverage than heterosexual males (25.4% and 32.6%, respectively). Bisexual females (33.9% and 58.5%, respectively) had higher HepA and HepB coverage than heterosexual females (23.5% and 38.4%, respectively) and higher HepB coverage than lesbian females (45.4%). Bisexual adults (34.1%) had lower influenza coverage than gay/lesbian (48.5%) and heterosexual adults (43.8%). Except for the association of having self-identified as gay/lesbian orientation with greater likelihood of HepA, HepB, and influenza vaccination, sexual orientation was not associated with higher or lower likelihood of vaccination. Health status or other behavioral characteristics studied had no consistent relationship with vaccination among all populations.

**Conclusion:**

Differences were identified in vaccination coverage among the U.S. adult population by self-reported sexual orientation. This study is the first to assess associations of sexual orientation with a comprehensive list of vaccinations. Findings from this study can serve as a baseline for monitoring changes over time. All populations could benefit from improved vaccination.

## Introduction

Comprehensive vaccination is critical in preventing the acquisition and transmission of many serious communicable infections [1–10]. Influenza vaccination is recommended for all adults each year. Other adult vaccinations are recommended for specific populations based on a person’s age, health conditions, behavioral risks, occupation, travel, and other indications (https://www.cdc.gov/vaccines/schedules/hcp/adult.html).

Differences in the receipt of select vaccines have been reported between persons who self-identify as lesbian, gay, or bisexual versus heterosexual populations [11–29]. Higher behavioral risks and lower rates of preventive care have been reported in lesbian and bisexual women compared with heterosexual women [30,31]. For example, prior surveillance has indicated that human papillomavirus (HPV) vaccination among sexual minority women (including lesbians, bisexual, and other women who have sex with women) remains low [23–25]. Sexual minority men, specifically men who identify as gay, bisexual, or as an MSM (men who have sex with men), have been reported to have an elevated risk for certain vaccine preventable infections, including infections due to HPV, hepatitis A virus (HAV), and hepatitis B virus (HBV), attributed to transmission through anal intercourse [1,2,32]. Disparities in HPV, hepatitis A (HepA), and hepatitis B (HepB) infections that burden sexual minority populations may be related to differential vaccination uptake compared with heterosexual adults [23–29]. With implementation of the Affordable Care Act, sexual minority adults experienced reduced barriers to accessing primary health care and health care affordability [30,33–37]. The extent to which health care providers can capitalize on the health care visits of sexual minority adults to increase comprehensive vaccination warrants exploration.

Limited research has evaluated the associations of sexual orientation with vaccination uptake [11–29]. Starting in 2013, the National Health Interview Survey (NHIS), which routinely conducts surveillance of vaccination histories, included questions to ascertain adult sexual orientation. Using data from the 2013, 2014, and 2015 NHIS cycles, this study examines vaccination differences among U.S. adults by their self-identified sexual orientation and explores factors associated with vaccination for selected vaccines. We hypothesized that self-identified gay/lesbian or bisexual populations will have higher coverage for select vaccines compared with heterosexual adults and there will be differences in coverage for select vaccines among sexual minorities. Because of greater use of health services among gay/lesbian adults and the generally higher acceptability of vaccines in these groups [29] and because HPV, hepatitis A (HepA), and hepatitis B (HepB) vaccinations protect against sexually transmitted infections, persons at higher risk of acquiring these infections because of risky sexual behaviors might be expected to have higher coverage for those vaccines.

## Material and methods

The NHIS was approved by the Research Ethics Review Board (ERB number, 2009–16) of the National Center for Health Statistics, CDC and designated as “Public Health Non-Research” during the determination for applicability of human subjects’ regulations, because the activity is not intended to include applicable research, but to access the implementation, coverage, performance, and/or satisfaction with an existing public health program, service, function, intervention or recommendation. Data security was addressed and written informed consent was sought.

### Survey description

The NHIS is a nationally representative probability-based health survey of the civilian noninstitutionalized U.S. population conducted continuously throughout the year by the National Center for Health Statistics of the Centers for Disease Control and Prevention [38]. The NHIS uses a complex sampling design involving stratification, clustering, and multistage sampling. Results obtained from samples are weighted to the adult civilian noninstitutionalized U.S. population to minimize non-response bias. Starting in 2013, questions allowing self-identification of sexual orientation were added to the survey. The adult questionnaire is completed by one randomly selected adult aged ≥18 years within each family in the household. This questionnaire included questions on receipt of recommended vaccines. Three years of data from 2013, 2014, and 2015 NHIS were combined to get an adequate sample size of lesbian, gay, and bisexual adults. The combined sample for this analysis was 101,091 adults aged ≥18 years. There are no unique personal identifiers in the NHIS that would allow determination of repeat respondents in this study across data recruitment waves. In 2013, 2014, and 2015, the final response rates among adults were 61.2%, 58.9%, and 55.2%, respectively [39–41].

### Sexual orientation assessment

To ascertain the adult’s self-identification of sexual orientation, four cascading questions were asked of all sample adults aged ≥18 years. The first question asked, “*Which of the following best represents how you think of yourself*?” The response options for males were: gay; straight, that is, not gay; bisexual; something else; and I don’t know the answer. The response options for females were: lesbian or gay; straight, that is, not lesbian or gay; bisexual; something else; and I don’t know the answer. In the 2013 and 2014 NHIS, respondents who answered “something else” or “I don’t know the answer” were asked one or more follow-up question(s) to gather additional information on their sexual orientation, while no follow-up question(s) were asked of respondents in the 2015 NHIS. Data from the 2013 and 2014 NHIS follow-up questions were not used in this study. Respondents answering “something else” (0.2%), “I don’t know the answer” (0.5%), and refused (0.5%) were classified as missing and excluded from the analysis.

### Vaccination variables

Responses to selected survey questions were used to measure associations between self-identified sexual orientation and self-reported receipt of selected vaccines (*HPV* [females and males aged 18–26 years], *HepA* [ever received; adult aged ≥18 years with high risk for HAV infection], *HepB* [ever received; adults aged ≥18 years with high risk for HBV infection], *influenza* [shot or spray], *pneumococcal* [adults aged 18–64 years at increased risk for pneumococcal disease and aged ≥65 years], *tetanus toxoid*, *reduced diphtheria toxoid*, *and acellular pertussis* vaccine [Tdap; analysis was limited to individuals who were able to say they received Td or Tdap during 2005 or later], and *shingles* vaccine [herpes zoster, adults aged ≥60 years]).

### Sociodemographic and health behavior variables

Sociodemographic and health behavior variables included: demographic characteristics (age, sex, race/ethnicity, education, employment, poverty status); access to healthcare (having health insurance, having a usual place for healthcare, physician contact within the past year); health behaviors (smoking status, consuming five or more alcoholic drinks in one day at least once in the past year, ever being tested for HIV, meeting federal guidelines for aerobic physical activity, self-selected health status); selected health conditions (asthma, diabetes, obesity, cardiovascular disease, chronic liver disease, disability); and travel status (ever traveled outside of the United States to countries other than Europe, Japan, Australia, New Zealand or Canada, since 1995 where HAV and/or HBV infections are endemic). Poverty status was defined according to the ratio of a family’s total income to the federal poverty threshold. Federal poverty thresholds are updated annually by the U.S. Census Bureau (https://www.census.gov/data/tables/time-series/demo/income-poverty/historical-poverty-thresholds.html).

### Increased risk for pneumococcal, hepatitis A, and hepatitis B infections

#### Pneumococcal disease

The presence of selected conditions that increase risk for pneumococcal disease was determined by responses to questions in the NHIS. Adults were considered at increased risk for pneumococcal disease if they had ever been told by a doctor or other health professional that they had diabetes, emphysema, chronic obstructive pulmonary disease, coronary heart disease, angina, heart attack, or other heart condition; had a diagnosis of cancer during the previous 12 months (excluding nonmelanoma skin cancer); had ever been told by a doctor or other health professional that they had lymphoma, leukemia, or blood cancer; or they had been told by a doctor or other health professional that they had chronic bronchitis or weak or failing kidneys during the preceding 12 months or had an asthma episode or attack during the preceding 12 months; or they were current smokers.

For HepA and HepB vaccination, data were collected on selected respondent characteristics that increase the risk for infection (travel to countries in which HAV infections are endemic and having chronic liver disease; travel to countries in which HBV infections are endemic, and having diabetes or chronic liver disease, respectively) [3,5].

#### Hepatitis A

All adults were considered at high-risk behavior for HAV infection if they reported any one of the following risk factors: ever traveled outside of the United States to countries other than Europe, Japan, Australia, New Zealand or Canada, since 1995 or if they had been ever told by a doctor; or other health professional that they had any kind of chronic, or long-term liver condition.

#### Hepatitis B

All adults were considered at high-risk behavior for HBV infection if they reported any one of the following risk factors: if they had ever been told by a doctor or other health professional that they had diabetes; ever traveled outside of the United States to countries other than Europe, Japan, Australia, New Zealand or Canada, since 1995; or if they had been ever told by a doctor or other health professional that they had any kind of chronic, or long-term liver condition.

### Data analysis

SUDAAN statistical software (Software for the statistical analysis of correlated data, Research Triangle Institute, Research Triangle Park, NC, version 11.0.1) was used to calculate point estimates and 95% confidence intervals for specified outcome variables. T-tests were used to determine differences between demographic and other characteristics stratified by sexual orientation. Statistical significance was defined as *p*<0.05. For vaccines other than influenza, the weighted proportion of respondents who reported vaccination was calculated. To better assess seasonal influenza vaccination coverage combined for the 2013–14 and 2014–15 influenza seasons, we reported coverage restricted to individuals interviewed during August 2013 through June 2014, and vaccinated during July 2013 through May 2014 for the 2013–14 influenza season, and individuals interviewed during August 2014 through June 2015, and vaccinated during July 2014 through May 2015 for the 2014–15 influenza season using the Kaplan-Meier survival analysis procedure [42–44].

Adjusted prevalence differences were estimated for each vaccine overall and stratified by sexual orientation using predictive marginals under multivariable logistic regression models. Adjusted estimates for the ‘*overall*’ model were produced by adjusting for age, sex, race/ethnicity, education, employment status, poverty status, physician contact within the past year, usual place of healthcare, health insurance status, self-reported smoking status, five or more alcoholic drinks in one day at least once in past year for men/four or more alcoholic drinks in one day a least once in the past year for women, HIV test status, self-reported health status, obesity status (defined as having body mass index ≥30), history of asthma, diabetes status, cardiovascular disease status, chronic liver disease status, disability status, met Federal guidelines for aerobic physical activity (using 2008 Guidelines criteria [45], respondents were classified as aerobically active if they reported ≥150 minutes/week of light- to moderate-intensity activity, 75 minutes/week of vigorous-intensity activity, or an equivalent combination of the two), and sexual orientation, and in addition, travel status for HAV and HBV vaccination coverage estimates. These variables were adjusted for in the multivariable logistic regression models, because these variables have been shown to be associated with vaccination.

Being the primary exposure of interest, sexual orientation was not controlled for except in the “*overall*” multivariable regression model(s). Adjusted estimates for the ‘stratified by sexual orientation’ model were produced by controlling for all variables adjusted for in the ‘*overall*’ model except the sexual orientation variable, which allowed reporting of adjusted vaccination coverage levels among the study population stratified on their sexual orientation. The adjusted prevalence differences (controlling for all variables included in the ‘*overall*’ model) were used to identify factors independently associated with vaccination, which allowed assessment of sexual orientation as an independent factor of vaccination.

## Results

Table 1 presents the percent distribution of sexual orientation by selected demographic, access to healthcare, health behavior, and health condition characteristics. Among adults aged ≥18 years, 97.6% self-identified as heterosexual, 1.6% as gay/lesbian, and 0.7% as bisexual. Respondents identifying as gay/lesbian and bisexual were more likely to report current smoking, consuming five or more alcoholic drinks in one day at least once in the past year, testing for HIV, and to have asthma compared with respondents identifying as heterosexual. Respondents identifying as gay/lesbian and bisexual differed from respondents identifying as heterosexual on a number of other demographic and access characteristics (Table 1).

**Table 1:** Characteristics of participants aged ≥18 years in the United States by demographic characteristics, health conditions, health behaviors, healthcare access, vaccination status, and self-identified sexual orientation–National Health Interview Survey, 2013–2015.

	Heterosexual		Gay/Lesbian		Bisexual	
	Sample	Weighted %	Sample	Weighted %	Sample	Weighted %
**Overall**	98,582	97.6	1,718	1.6	791	0.7
**Age group (years)**						
18–49	49,707	55.1	1,063	**64.5**^a^	626	**81.6**^**b**^^,^^**c**^
50–64	25,231	25.9	468	26.4	113	**12.9**^**b**^^,^^**c**^
65 and over	23,644	19.0	187	**9.1**^a^	52	**5.5**^**b**^^,^^**c**^
**Sex**						
Male	44,057	48.3	930	**54.6**^a^	237	**29.0**^**b**^^,^^**c**^
Female	54,525	51.7	788	45.4	554	71.0
**Race/ethnicity**						
Non-Hispanic white	60,449	65.6	1,085	66.4	513	69.7
Non-Hispanic black	13,504	11.5	242	12.7	104	10.5
Hispanic	16,565	15.3	266	14.2	111	**11.9**^**b**^
Non-Hispanic other, multiple races	8,064	7.5	125	6.7	63	7.9
**Education status**						
Less than HS	14,604	13.2	125	**6.7**^**a**^	93	**14.6**^**b**^^,^^**c**^
HS graduate	25,309	25.7	315	**19.4**^**a**^	160	**20.9**^**b**^
College and above	58,263	61.0	1,276	**74.0**^**a**^	538	**64.5**^**c**^
**Employment status**						
Employed	57,091	61.1	1,183	**68.2**^**a**^	500	**60.0**^**c**^
Unemployed	4,305	4.7	111	6.1	80	**12.8**^**b**^^,^^**c**^
Not in work force	37,150	34.2	423	**25.7**^**a**^	211	**27.2**^**b**^
**Poverty status**^**d**^						
At or above poverty	76,884	82.1	1,405	**84.7**^**a**^	540	**73.2**^**b**^^,^^**c**^
Below poverty	15,796	12.3	265	12.5	220	**23.3**^**b**^^,^^**c**^
Unknown	5,212	5.6	40	**2.7**^**a**^	25	**3.5**^**b**^
**Physician contact within past year**						
≥1	80,377	81.4	1,418	81.6	655	82.1
0	17,903	18.6	294	18.4	134	17.9
**Has a usual place to go for healthcare**						
Yes	84,632	85.9	1,441	85.0	643	**79.4**^**b**^^,^^**c**^
No	13,926	14.1	276	15.0	148	20.6
**Has health insurance**						
Yes	84,821	86.6	1,465	85.7	655	**82.4**^**b**^
No	13,403	13.4	241	14.3	131	17.6
**Smoking status**						
Current cigarette smoker	16,726	16.4	423	**22.8**^**a**^	225	**25.4**^**b**^
Former cigarette smoker	22,406	22.0	408	22.9	163	18.6
Never cigarette smoker	59,304	61.6	884	**54.3**^**a**^	403	**56.0**^**b**^
**Five or more alcoholic drinks in 1 day at least once in past year**						
Yes	21,641	24.8	573	**34.2**^**a**^	338	**42.9**^**b**^^,^^**c**^
No	69,121	75.2	1,034	65.8	409	57.1
**Ever been tested for HIV**						
Yes	36,599	37.2	1,177	**67.7**^**a**^	487	**58.8**^**b**^^,^^**c**^
No	59,702	62.8	521	32.3	294	41.2
**Self-selected health status**						
Excellent/very good	56,902	60.8	1,068	62.1	438	56.2
Other	41,640	39.2	650	37.9	353	43.8
**Obesity status**						
Yes	28,684	29.3	495	29.5	271	**36.0**^**b**^^,^^**c**^
No	67,207	70.7	1,200	70.5	505	64.0
**Asthma**						
Yes	3,641	3.4	104	**5.6**^**a**^	66	**7.3**^**b**^
No	94,854	96.6	1,612	94.4	725	92.7
**Diabetes**						
Yes	10,467	9.4	137	**7.1**^**a**^	47	**5.8**^**b**^
No	88,065	90.6	1,580	92.9	743	94.2
**Cardiovascular disease**						
Yes	12,668	11.6	184	10.5	79	**8.6**^**b**^
No	85,786	88.4	1,533	89.5	712	91.4
**Chronic liver disease**						
Yes	1,304	1.2	38	1.7	21	1.9
No	97,188	98.8	1,677	98.3	770	98.1
**Disability status**						
Yes	47,835	46.4	989	**55.7**^**a**^	552	**69.3**^**b**^^,^^**c**^
No	50,747	53.6	729	44.3	239	30.7
**Met federal guidelines for aerobic physical activity**						
Yes	8,069	8.7	137	7.9	65	6.9
No	89,812	91.3	1,566	92.1	717	93.1
**Travel**^**e**^						
Yes	32,603	35.0	635	37.5	270	34.1
No	65,882	65.0	1,082	62.5	521	65.9

**Note:** Boldface indicates significance.

^a^p<0.05 by t-test for comparisons between heterosexual and gay/lesbian within each level of characteristic.

^b^p<0.05 by t-test for comparisons between heterosexual and bisexual within each level of characteristic.

^**c**^p<0.05 by t-test for comparisons between gay/lesbian and bisexual within each level of characteristic.

^d^Poverty status was defined according to the ratio of a family’s total income to the federal poverty threshold. Federal poverty thresholds are updated annually by the U.S. Census Bureau (https://www.census.gov/data/tables/time-series/demo/income-poverty/historical-poverty-thresholds.html).

^e^Persons from developed countries who travel to developing countries with high or intermediate hepatitis A virus [HAV] and hepatitis B virus [HBV] endemicity are considered at substantial risk for acquiring HAV and HBV infections. Persons who traveled outside the United States to countries other than Europe, Japan, Australia, New Zealand, or Canada were considered having traveled to countries with high or intermediate HAV and HBV endemicity.

Table 2 presents the unadjusted self-reported vaccination coverage of selected vaccines among adults aged ≥18 years, overall and by sexual orientation. Overall HPV vaccination coverage (receipt of at least one dose) among adults aged 18–26 years was 23.5%. Coverage among males and females was 9.5% and 37.9%, respectively. HPV vaccination coverage among bisexual females (51.6%) was significantly higher compared with heterosexual females (40.2%). Overall, HepA vaccination coverage (ever received) among adults aged ≥18 years at high risk for HAV infection was 24.7%, and was significantly higher among gay/lesbian (35.1%) and bisexual (38.0%) compared with heterosexual adults (24.5%). Overall, HepA vaccination coverage among males aged ≥18 years was 25.7%, and was significantly higher among gay males (40.3%) compared with heterosexual males (25.4%). Overall HepA vaccination coverage among females aged ≥18 years was 23.7%, and was significantly higher among bisexual females (33.9%) compared with heterosexual females (23.5%). Overall HepB vaccination coverage (ever received) among adults aged ≥18 years at high risk for HBV infection was 35.8%; HepB vaccination coverage among gay/lesbian (49.8%) and bisexual adults (55.1%) was significantly higher compared with heterosexual adults (35.5%). Overall HepB vaccination coverage among males aged ≥18 years was 32.9% and coverage among gay males (52.6%) was significantly higher than among heterosexual males (32.6%). Overall HepB vaccination coverage among females aged ≥18 years was 38.6% and coverage among bisexual females (58.5%) was significantly higher than among heterosexual females (38.4%) and lesbian females (45.4%). Influenza vaccination coverage overall in the combined 2013–14 and 2014–15 seasons among adults aged ≥18 years was 43.8% and influenza vaccination coverage among bisexual adults (34.1%) was significantly lower compared with heterosexual (43.8%) and gay/lesbian adults (48.5%). Overall Tdap vaccination coverage among adults aged ≥18 years was 20.3% and was significantly higher among gay/lesbian (24.2%) and bisexual (29.8%) compared with heterosexual adults (20.3%). Overall pneumococcal vaccination coverage among adults aged 18–64 years at increased risk for pneumococcal disease was 20.4% and was similar across sexual orientation categories. Pneumococcal vaccination coverage overall among adults aged ≥65 years was 59.2% and was similar across sexual orientation categories. Shingles coverage overall among adults aged ≥60 years was 26.8% and was similar across sexual orientation categories (Table 2).

**Table 2:** Unadjusted vaccination coverage of selected vaccines among adults aged ≥18 years in the United States by self-identified sexual orientation–National Health Interview Survey, 2013–2015.

			Sexual Orientation					
	Overall		Heterosexual		Gay/Lesbian		Bisexual	
	n	% (95% CI)	n	% (95% CI)	n	% (95% CI)	n	% (95% CI)
Human papillomavirus vaccination (≥1 dose)								
18–26 years	13,065	23.5 (22.3, 24.7)	12,083	24.9 (23.6, 26.2)	294	28.6 (21.6, 36.8)	237	**42.8 (34.4, 51.7)**^**a**^^,^^**b**^
Male	6,267	9.5 (8.4, 10.6)	5,854	10.0 (8.8, 11.4)	146	17.9 (10.7, 28.4)	62	15.3 (6.9, 30.8)
Female	6,798	37.9 (36.0, 39.7)	6,229	40.2 (38.3, 42.2)	148	40.8 (29.8, 52.8)	175	**51.6 (41.5, 61.6)**^**a**^
Hepatitis A vaccination (ever received)								
18+ years high-risk^d^	31,042	24.7 (24.0, 25.4)	29,710	24.5 (23.8, 25.2)	587	**35.1 (30.7, 39.6)**^**a**^	245	**38.0 (30.6, 45.9)**^**a**^
18+ years high-risk, male	14,725	25.7 (24.7, 26.8)	14,062	25.4 (24.4, 26.4)	368	**40.3 (34.1, 46.8)**^**a**^	70	NR^c^
18+ years high-risk, female	16,317	23.7 (22.8, 24.6)	15,648	23.5 (22.6, 24.5)	219	27.0 (20.1, 35.2)	175	**33.9 (25.6, 43.3)**^**a**^
Hepatitis B vaccination (ever received)								
18+ years high-risk^e^	39,939	35.8 (35.0, 36.5)	38,290	35.5 (34.8, 36.2)	696	**49.8 (44.9, 54.6)**^**a**^	290	**55.1 (47.1, 62.7)**^**a**^
18+ years high-risk, male	18,618	32.9 (32.0, 33.9)	17,821	32.6 (31.6, 33.6)	428	**52.6 (45.9, 59.3)**^**a**^	85	NR^c^
18+ years high-risk, female	21,321	38.6 (37.7, 39.5)	20,469	38.4 (37.4, 39.3)	268	45.4 (37.5, 53.6)	205	**58.5 (48.8, 67.5)**^**a**^^,^^**b**^
Influenza vaccination^**f**^								
18+ years	64,855	43.8 (42.9, 44.7)	61,968	43.8 (42.9, 44.7)	1,074	48.5 (42.4, 55.0)	512	**34.1 (26.9, 42.6)**^**a**^^,^^**b**^
Tdap vaccination								
18+ years	66,380	20.3 (19.8, 20.9)	63,505	20.3 (19.8, 20.9)	1,113	**24.2 (21.2, 27.6)**^**a**^	525	**29.8 (24.3, 35.8)**^**a**^
Pneumococcal vaccination (ever received)								
18–64 years increased risk^g^	28,000	20.4 (19.8, 21.1)	25,973	21.2 (20.5, 21.9)	654	25.2 (20.8, 30.1)	337	21.4 (15.0, 29.6)
65+ years	24,754	59.2 (58.4, 60.0)	23,644	61.7 (60.8, 62.6)	187	63.3 (53.2, 72.4)	52	NR^c^
Shingles vaccination (ever received)								
60+ years	33,271	26.8 (26.0, 27.6)	31,675	27.8 (26.9, 28.6)	296	30.4 (23.9, 37.9)	84	NR^c^

*CI* confidence interval.

**Note:** Boldface indicates significance.

^a^p<0.05 by t-test (comparing against heterosexual).

^b^p<0.05 by t-test (comparing against gay/lesbian).

^c^Estimate is not reported because it is unreliable either due to effective small sample size (n<30) and/or CI half-width >15 as per the National Center for Health Statistics Data Presentation Standards for Proportions, Series 2 Report available at: https://www.cdc.gov/nchs/data/series/sr_02/sr02_175.pdf.

^d^Adults were considered at high-risk for hepatitis A virus (HAV) infection if they reported any one of the following risk factors: ever traveled outside of the United States to countries other than Europe, Japan, Australia, New Zealand or Canada, since 1995 or if they had been ever told by a doctor; or other health professional that they had any kind of chronic, or long-term liver condition.

^e^Adults were considered at high-risk for hepatitis B virus (HBV) infection if they reported any one of the following risk factors: if they had ever been told by a doctor or other health professional that they had diabetes; ever traveled outside of the United States to countries other than Europe, Japan, Australia, New Zealand or Canada, since 1995; or if they had been ever told by a doctor or other health professional that they had any kind of chronic, or long-term liver condition.

^f^Influenza vaccination coverage estimates represent the coverage for an influenza season calculated using the Kaplan-Meier method. The calculation includes respondents vaccinated July 2013 to May 2014 and interviewed August 2013 to June 2014, and July 2014 to May 2015 and interviewed August 2014 to June 2015.

^g^Adults were considered at increased risk for pneumococcal disease if they had ever been told by a doctor or other health professional that they had diabetes, emphysema, chronic obstructive pulmonary disease, coronary heart disease, angina, heart attack, or other heart condition; had a diagnosis of cancer during the previous 12 months (excluding nonmelanoma skin cancer); had ever been told by a doctor or other health professional that they had lymphoma, leukemia, or blood cancer; or they had been told by a doctor or other health professional that they had chronic bronchitis or weak or failing kidneys during the preceding 12 months or had an asthma episode or attack during the preceding 12 months; or they were current smokers.

Adjusted vaccination coverage for each vaccine class is shown in Table 3, overall and stratified by each sexual orientation category. Overall adjusted HepA vaccination coverage among gay/lesbian and bisexual adults was significantly higher compared with heterosexual adults. Adjusted HepA vaccination coverage was significantly higher among gay males compared with heterosexual males. Overall adjusted HepB vaccination coverage among gay/lesbian and bisexual adults was significantly higher compared with heterosexual adults. Adjusted HepB vaccination coverage was significantly higher among gay males compared with heterosexual males. After adjustment, overall influenza vaccination coverage was significantly higher among gay/lesbian compared with heterosexual adults, but significantly lower among bisexual compared with gay/lesbian adults. There were no significant differences by sexual orientation for Tdap, pneumococcal (among adults aged 18–64 years with high-risk conditions and those aged ≥65 years) or shingles vaccination (Table 3).

**Table 3:** Adjusted vaccination coverage of selected vaccines among adults aged ≥18 years in the United States by self-identified sexual orientation–National Health Interview Survey, 2013–2015.

				Sexual Orientation					Prevalence Difference (PD) (adjusted)^a^					
		Overall^b^		Heterosexual		Gay or Lesbian		Bisexual	Gay/Lesbian vs. Heterosexual		Bisexual vs. Heterosexual		Bisexual vs. Gay/Lesbian	
	n	% (95% CI)	n	% (95% CI)	n	% (95% CI)	n	% (95% CI)	% (95% CI)	*p*-Value	% (95% CI)	*p*-Value	% (95% CI)	*p*-Value
Human papillomavirus vaccination (≥1 dose)														
18–26 years	13,065	25.5 (24.2, 26.8)	11,453	25.2 (23.8, 26.6)	280	27.8 (20.7, 36.2)	226	39.8 (32.1, 48.2)	2.6 (-5.4, 10.6)	0.523	**14.7 (6.4, 22.9)**^**c**^	**<0.001**	**12.1 (0.6, 23.5)**^**d**^	**0.040**
Male	6,267	10.3 (9.2, 11.7)	5,500	10.1 (8.9, 11.5)	136	16.0 (9.5, 25.8)	58	18.6 (8.4, 36.4)	5.9 (-2.2, 14.0)	0.153	8.5 (-5.3, 22.3)	0.229	NR^e^	-
Female	6,798	41.0 (39.0, 43.1)	5,953	40.6 (38.6, 42.8)	144	45.4 (33.8, 57.6)	168	50.8 (40.4, 61.1)	4.8 (-7.5, 17.0)	0.446	10.1 (-0.6, 20.8)	0.065	NR^e^	—
Hepatitis A vaccination (ever received)														
18+ years high-risk^f^	31,042	24.9 (24.1, 25.7)	29,710	24.7 (23.9, 25.5)	587	30.9 (27.0, 35.2)	245	32.8 (26.0, 40.4)	**6.2 (2.1, 10.3)**^**c**^	**0.003**	**8.1 (0.9, 15.3)**^**c**^	**0.03**	1.9 (-6.3, 10.1)	0.655
18+ years high-risk, male	15,342	25.7 (24.6, 26.8)	14,062	25.6 (24.5, 26.7)	368	32.6 (27.1, 38.6)	70	NR^e^	**6.9 (1.2, 12.7)**^**c**^	**0.02**	NR^e^	-	NR^e^	-
18+ years high-risk, female	17,132	23.6 (22.6, 24.6)	15,648	23.8 (22.8, 24.8)	219	27.1 (20.0, 35.6)	175	28.9 (21.2, 37.9)	3.3 (-4.6, 11.2)	0.417	5.1 (-3.4, 13.5)	0.239	1.8 (-8.8, 12.4)	0.742
Hepatitis B vaccination (ever received)														
18+ years high-risk^g^	39,939	36.4 (35.6, 37.2)	38,290	36.2 (35.4, 37.0)	696	43.6 (38.9, 48.5)	290	44.0 (36.7, 51.6)	**7.4 (2.6, 12.3)**^**c**^	**0.003**	**7.8 (0.3, 15.2)**^**c**^	**0.042**	0.3 (-8.6, 9.3)	0.939
18+ years high-risk, male	19,204	33.7 (32.7, 34.8)	17,821	33.3 (32.3, 34.4)	428	44.5 (38.4, 50.8)	85	NR^e^	**11.2 (4.9, 17.5)**^**c**^	**<0.001**	NR^e^	-	NR^e^	-
18+ years high-risk, female	22,105	39.3 (38.3, 40.3)	20,469	39.1 (38.1, 40.2)	268	40.1 (32.3, 48.3)	205	47.2 (38.0, 56.7)	0.9 (-7.1, 8.9)	0.821	8.1 (-1.4, 17.6)	0.096	7.2 (-5.5, 19.8)	0.266
Influenza vaccination														
18+ years	64,855	42.0 (41.3, 42.8)	61,968	42.0 (41.2, 42.8)	1,074	48.1 (44.2, 52.0)	512	37.1 (30.7, 43.9)	**6.1 (2.3, 10.0)**^**c**^	**0.002**	-4.9 (-11.5, 1.8)	0.150	**-11.0 (-18.3, -3.7)**^**d**^	**0.003**
Tdap vaccination														
18+ years	66,380	20.9 (20.4, 21.5)	63,505	20.9 (20.3, 21.5)	1,113	20.7 (17.9, 23.9)	525	25.3 (20.4, 30.9)	-0.2 (-3.1, 2.8)	0.916	4.4 (-0.8, 9.6)	0.101	4.6 (-1.3, 10.4)	0.127
Pneumococcal vaccination (ever received)														
18–64 years increased risk^h^	28,000	20.8 (20.1, 21.6)	25,973	20.7 (20.0, 21.5)	654	24.2 (20.2, 28.6)	337	22.5 (16.8, 29.5)	3.5 (-0.8, 7.7)	0.110	1.8 (-4.7, 8.2)	0.591	-1.7 (-9.0, 5.6)	0.649
65+ years	24,754	61.8 (60.8, 62.8)	23,644	61.8 (60.8, 62.8)	187	58.5 (48.9, 67.5)	52	NR^e^	-3.3 (-12.7, 6.1)	0.492	NR^e^	-	NR^e^	-
Shingles vaccination (ever received)														
60+ years	33,271	28.5 (27.6, 29.5)	31,675	28.5 (27.6, 29.5)	296	25.8 (20.0, 32.7)	84	NR^e^	-2.7 (-9.1, 3.7)	0.413	NR^e^	-	NR^e^	-

*CI* confidence interval.

**Note:** Boldface indicates significance.

^a^Adjusted estimates control for age, sex, race/ethnicity, education, employment status, poverty status, physician contact within the past year, usual place of healthcare, health insurance status, self-reported smoking status, five or more alcoholic drinks in 1 day at least once in past year, HIV test status, self-reported health status, obesity status, asthma status, diabetes status, cardiovascular disease status, chronic liver disease status, disability status, and met Federal guidelines for aerobic physical activity, and in addition, *travel for hepatitis A and hepatitis B vaccination*.

^b^In addition to all other noted adjustment factors, overall estimates also controlled for self-reported sexual orientation.

^c^p<0.05 by t-test (comparing against heterosexual).

^d^p<0.05 by t-test (comparing against gay/lesbian).

^e^Estimate is not reported because it is unreliable either due to effective small sample size (n<30) and/or CI half-width >15 as per the National Center for Health Statistics Data Presentation Standards for Proportions, Series 2 Report available at: https://www.cdc.gov/nchs/data/series/sr_02/sr02_175.pdf.

^f^Adults were considered at high-risk for hepatitis A virus (HAV) infection if they reported any one of the following risk factors: ever traveled outside of the United States to countries other than Europe, Japan, Australia, New Zealand or Canada, since 1995 or if they had been ever told by a doctor; or other health professional that they had any kind of chronic, or long-term liver condition.

^g^Adults were considered at high-risk for hepatitis B virus (HBV) infection if they reported any one of the following risk factors: if they had ever been told by a doctor or other health professional that they had diabetes; ever traveled outside of the United States to countries other than Europe, Japan, Australia, New Zealand or Canada, since 1995; or if they had been ever told by a doctor or other health professional that they had any kind of chronic, or long-term liver condition.

^h^Adults were considered at increased risk for pneumococcal disease if they had ever been told by a doctor or other health professional that they had diabetes, emphysema, chronic obstructive pulmonary disease, coronary heart disease, angina, heart attack, or other heart condition; had a diagnosis of cancer during the previous 12 months (excluding nonmelanoma skin cancer); had ever been told by a doctor or other health professional that they had lymphoma, leukemia, or blood cancer; or they had been told by a doctor or other health professional that they had chronic bronchitis or weak or failing kidneys during the preceding 12 months or had an asthma episode or attack during the preceding 12 months; or they were current smokers.

Table 4 presents the results of the multivariable logistic regression models by selected vaccines. Except for the association of having self-identified as gay/lesbian orientation with greater likelihood of HepA, HepB, and influenza vaccination, sexual orientation was not associated with higher or lower likelihood of vaccination. Health status or other behavioral characteristics studied had no consistent relationship with vaccination among all populations. After controlling for sexual orientation, other characteristics that are generally associated with vaccination were still independently associated with likelihood of vaccination (Table 4).

**Table 4:** Multivariable logistic regression analysis among persons aged ≥18 years in the United States who reported receiving selected vaccines by self-identified sexual orientation, demographic characteristics, health conditions, health behaviors, and healthcare access–National Health Interview Survey, 2013–2015.

	Human Papillomavirus Vaccination (≥1 dose)	Hepatitis A Vaccination (ever received)	Hepatitis B Vaccination (ever received)	Influenza Vaccination	Tdap Vaccination	Shingles Vaccination (ever received)	Pneumococcal Vaccination (ever received)	
	18–26 years (n = 13,065)	18 and over high-risk^a^ (n = 31,042)	18 and over high-risk^b^ (n = 39,939)	18 and over (n = 64,855)	18 and over (n = 66,380)	60 and over (n = 33,271)	18–64 increased risk^c^ (n = 28,000)	65 and over (n = 24,754)
	APD^d^	APD^e^	APD^e^	APD^d^	APD^d^	APD^d^	APD^d^	APD^d^
	(95% CI)	(95% CI)	(95% CI)	(95% CI)	(95% CI)	(95% CI)	(95% CI)	(95% CI)
**Sexual Orientation**								
Heterosexual	Referent	Referent	Referent	Referent	Referent	Referent	Referent	Referent
Gay/lesbian	5.9 (-1.4, 13.2)	**6.2 (2.1, 10.3)**	**7.4 (2.6, 12.3)**	**6.1 (2.2, 10.0)**	-0.2 (-3.1, 2.8)	-2.7 (-9.1, 3.7)	3.5 (-0.8, 7.7)	-3.3 (-12.7, 6.1)
Bisexual	7.0 (-0.2, 14.2)	**8.1 (0.9, 15.3)**	**7.8 (0.3, 15.2)**	-5.0 (-11.6, 1.7)	4.4 (-0.8, 9.6)	NR^f^	1.8 (-4.7, 8.2)	NR^f^
**Age group (years)**								
18–49	NA	Referent	Referent	Referent	NA	NA	Referent	NA
50–64	NA	**-10.1 (-11.7, -8.5)**	**-12.3 (-14.1, -10.5)**	**9.6 (8.1, 11.1)**	NA	NA	**9.6 (8.1, 11.1)**	NA
65 and over	NA	**-12.9 (-15.3, -10.6)**	**-22.5 (-24.5, -20.4)**	**26.2 (24.2, 28.2)**	NA	NA	NA	NA
18–21	Referent	NA	NA	NA	NA	NA	NA	NA
22–26	**-11.1 (-13.7, -8.6)**	NA	NA	NA	NA	NA	NA	NA
18–24	NA	NA	NA	NA	Referent	NA	NA	NA
25–34	NA	NA	NA	NA	**-4.6 (-6.7, -2.4)**	NA	NA	NA
35–44	NA	NA	NA	NA	**-10.4 (-12.7, -8.1)**	NA	NA	NA
45–64	NA	NA	NA	NA	**-49.1 (-51.3, -46.9)**	NA	NA	NA
65 and over	NA	NA	NA	NA	**-45.5 (-47.7, -43.3)**	NA	NA	NA
60–64	NA	NA	NA	NA	NA	Referent	NA	NA
65–74	NA	NA	NA	NA	NA	**10.5 (8.6, 12.5)**	NA	Referent
75–84	NA	NA	NA	NA	NA	**9.0 (6.8, 11.1)**	NA	**8.5 (6.3, 10.6)**
85 and over	NA	NA	NA	NA	NA	**-49.8 (-52.7, -46.9)**	NA	**10.0 (7.1, 13.0)**
**Sex**								
Male	Referent	NA	NA	Referent	Referent	Referent	Referent	Referent
Female	**28.1 (25.6, 30.5)**	**-3.3 (-4.7, -1.8)**	**5.3 (4.0, 6.6)**	**3.7 (2.6, 4.7)**	**1.2 (0.1, 2.2)**	**4.7 (3.2, 6.3)**	0.0 (-1.3, 1.4)	**5.8 (3.9, 7.7)**
**Race/ethnicity**								
Non-Hispanic white	Referent	Referent	Referent	Referent	Referent	Referent	Referent	Referent
Non-Hispanic black	**-3.9 (-7.3, -0.6)**	**-3.3 (-6.1, -0.5)**	**-3.1 (-5.6, -0.7)**	**-7.3 (-8.9, -5.6)**	**-10.3 (-11.7, -9.0)**	**-17.3 (-19.4, -15.1)**	**-3.8 (-5.6, -2.0)**	**-16.2 (-18.9, -13.6)**
Hispanic	-2.0 (-4.6, 0.6)	**-4.2 (-6.2, -2.1)**	**-5.1 (-7.0, -3.2)**	**-4.0 (-5.8, -2.1)**	**-8.4 (-9.8, -7.0)**	**-12.6 (-15.2, -10.0)**	**-3.6 (-5.5, -1.7)**	**-17.4 (-20.6, -14.3)**
Non-Hispanic other, multiple races	-2.3 (-6.1, 1.4)	2.1 (-0.2, 4.5)	**2.6 (0.4, 4.8)**	**2.3 (0.3, 4.3)**	**-4.1 (-6.1, -2.2)**	**-8.0 (-11.0, -4.9)**	**-3.5 (-6.1, -1.0)**	**-12.1 (-15.8, -8.4)**
**Education status**								
Less than HS	Referent	Referent	Referent	Referent	Referent	Referent	Referent	Referent
HS graduate	-0.1 (-3.8, 3.6)	2.2 (-0.8, 5.3)	2.5 (-0.4, 5.4)	-1.4 (-3.4, 0.7)	0.8 (-0.9, 2.4)	**6.0 (3.8, 8.3)**	2.0 (-0.0, 4.0)	**4.1 (1.6, 6.5)**
College and above	**5.6 (2.0, 9.1)**	**10.6 (7.7, 13.5)**	**14.4 (11.6,17.1)**	**4.7 (2.8, 6.5)**	**8.2 (6.6, 9.8)**	**14.0 (11.9, 16.2)**	**3.7 (1.7, 5.7)**	**7.4 (4.9, 9.9)**
**Employment status**								
Employed	Referent	Referent	Referent	Referent	Referent	Referent	Referent	Referent
Unemployed	-0.8 (-4.6, 3.0)	3.2 (-0.4, 6.8)	1.3 (-2.1, 4.8)	**-5.1 (-8.2, -2.1)**	-1.2 (-3.5, 1.0)	**-12.3 (-17.6, -6.9)**	**3.3 (0.0, 6.5)**	0.2 (-12.1, 12.5)
Not in work force	-1.7 (-4.6, 1.2)	**5.0 (3.0, 7.0)**	-0.4 (-2.2, 1.4)	**2.0 (0.4, 3.5)**	0.9 (-0.4, 2.2)	**3.5 (1.8, 5.1)**	**8.1 (6.3, 9.9)**	**9.0 (6.4, 11.7)**
**Poverty status**^**g**^								
Below poverty	Referent	Referent	Referent	Referent	Referent	Referent	Referent	Referent
At or above poverty	-1.1 (-4.0, 1.7)	**-4.0 (-6.8, -1.2)**	**-3.1 (-5.5, -0.6)**	**4.1 (2.4, 5.8)**	**3.6 (2.2, 5.1)**	**8.1 (5.7, 10.5)**	0.2 (-1.7, 2.1)	**8.8 (5.6, 12.0)**
Unknown	-4.9 (-11.1, 1.3)	**-6.3 (-10.7, -2.0)**	**-4.4 (-8.4, -0.4)**	1.7 (-1.5, 4.8)	-2.0 (-4.4, 0.5)	**5.1 (1.4, 8.7)**	-0.8 (-4.3, 2.7)	**5.8 (1.5, 10.2)**
**Physician contact within past year**								
0	Referent	Referent	Referent	Referent	Referent	Referent	Referent	Referent
≥1	**7.8 (5.2, 10.5)**	**4.7 (3.0,6.4)**	**4.6 (2.8, 6.4)**	**16.0 (14.3, 17.7)**	**8.7 (7.6, 9.9)**	**13.8 (11.4, 16.2)**	**5.4 (3.5, 7.3)**	**22.8 (18.5, 27.0)**
**Has a usual place to go for healthcare**								
No	Referent	Referent	Referent	Referent	Referent	Referent	Referent	Referent
Yes	**3.6 (1.1, 6.2)**	**-2.7 (-4.9, -0.5)**	**-2.5 (-4.7, -0.3)**	**10.3 (8.4, 12.2)**	**4.3 (2.8, 5.8)**	**12.1 (8.3, 15.9)**	**4.7 (2.5, 6.9)**	**20.5 (15.0, 26.1)**
**Has health insurance**								
No	Referent	Referent	Referent	Referent	Referent	Referent	Referent	Referent
Yes	**10.4 (7.5, 13.2)**	2.5 (-0.0, 5.0)	**5.8 (3.4, 8.3)**	**13.2 (11.3, 15.0)**	**5.2 (3.8, 6.7)**	**8.6 (2.7, 14.5)**	**4.0 (2.2, 5.8)**	**18.3 (6.9, 29.7)**
**Smoking status**								
Never smoker	Referent	Referent	Referent	Referent	Referent	Referent	Referent	Referent
Current smoker	1.6 (-2.0, 5.2)	-2.0 (-4.3, 0.3)	**-3.5 (-5.8, -1.1)**	**-8.8 (-10.5, -7.1)**	**-2.8 (-4.2, -1.5)**	**-7.8 (-10.5, -5.2)**	**-2.3 (-3.9, -0.6)**	-0.9 (-4.3, 2.5)
Former smoker	-2.7 (-6.7, 1.2)	**-2.3 (-4.0, -0.6)**	**-2.8 (-4.5, -1.1)**	1.3 (-0.2, 2.7)	-0.7 (-1.9, 0.4)	1.5 (-0.1, 3.1)	**3.5 (1.3, 5.7)**	**3.8 (1.8, 5.7)**
**Five or more alcoholic drinks in 1 day at least once in past year**								
No	Referent	Referent	Referent	Referent	Referent	Referent	Referent	Referent
Yes	2.8 (-1.5, 7.2)	0.4 (-2.0, 2.7)	0.4 (-1.8, 2.7)	**-3.8 (-5.6, -2.0)**	**1.7 (0.2, 3.3)**	2.4 (-0.6, 5.4)	**-5.3 (-7.3, -3.4)**	-3.5 (-7.3, 0.4)
**Ever been tested for HIV**								
No	Referent	Referent	Referent	Referent	Referent	Referent	Referent	Referent
Yes	**5.7 (3.2, 8.1)**	**10.2 (8.8, 11.7)**	**13.0 (11.4, 14.5)**	**4.0 (2.8, 5.2)**	**7.4 (6.4, 8.4)**	**3.9 (1.7, 6.0)**	**4.3 (2.9, 5.8)**	**5.2 (2.8, 7.6)**
**Self-selected health status**								
Other	Referent	Referent	Referent	Referent	Referent	Referent	Referent	Referent
Excellent/ good	1.2 (-1.2, 3.6)	**2.8 (1.3, 4.3)**	**2.0 (0.4, 3.5)**	-0.7 (-2.1, 0.6)	**2.8 (1.7, 3.9)**	**4.4 (2.8, 6.1)**	**-3.5 (-5.0, -2.0)**	0.0 (-2.1, 2.2)
**Obesity status**								
No	Referent	Referent	Referent	Referent	Referent	Referent	Referent	Referent
Yes	**-3.2 (-6.1, -0.4)**	**-2.5 (-4.3, -0.8)**	-0.8 (-2.3, 0.8)	1.0 (-0.4, 2.3)	**1.8 (0.7, 2.9)**	0.4 (-1.2, 1.9)	1.1 (-0.3, 2.6)	**2.5 (0.2, 4.7)**
**Asthma**								
No	Referent	Referent	Referent	Referent	Referent	Referent	Referent	Referent
Yes	2.6 (-2.5, 7.6)	1.6 (-2.3, 5.6)	3.2 (-0.3, 6.7)	2.4 (-0.7, 5.4)	**6.6 (4.1, 9.1)**	1.6 (-2.4, 5.6)	**9.7 (7.2, 12.3)**	**11.3 (6.4, 16.2)**
**Diabetes**								
No	Referent	Referent	Referent	Referent	Referent	Referent	Referent	Referent
Yes	-0.8 (-11.0, 9.4)	-1.1 (-3.7, 1.5)	-0.5 (-3.6, 2.7)	**6.9 (5.0, 8.9)**	-0.5 (-2.2, 1.3)	-1.2 (-3.1, 0.7)	**11.9 (9.8, 14.0)**	**7.9 (5.8, 10.1)**
**Cardiovascular disease**								
No	Referent	Referent	Referent	Referent	Referent	Referent	Referent	Referent
Yes	0.0 (-5.4, 5.4)	0.8 (-2.0, 3.5)	1.8 (-0.6, 4.2)	**3.5 (1.7, 5.3)**	**2.0 (0.3, 3.6)**	-0.4 (-2.0, 1.1)	**3.3 (1.6, 4.9)**	**8.5 (6.4, 10.6)**
**Chronic liver disease**								
No	Referent	Referent	Referent	Referent	Referent	Referent	Referent	Referent
Yes	NR**	0.5 (-6.4, 7.4)	**8.4 (3.4, 13.4)**	**5.4 (0.4, 10.4)**	1.4 (-2.5, 5.3)	0.3 (-5.9, 6.5)	**7.1 (3.2, 11.1)**	-5.3 (-13.1, 2.5)
**Disability status**								
No	Referent	Referent	Referent	Referent	Referent	Referent	Referent	Referent
Yes	3.4 (-1.0, 7.8)	-0.1 (-2.2, 2.0)	-0.1 (-2.1, 1.9)	0.6 (-0.9, 2.1)	-1.0 (-2.4, 0.3)	**-3.1 (-4.8, -1.5)**	**4.5 (2.6, 6.3)**	**3.0 (0.9, 5.0)**
**Met federal guidelines for aerobic physical activity**								
No	Referent	Referent	Referent	Referent	Referent	Referent	Referent	Referent
Yes	1.3 (-1.7, 4.4)	**2.3 (0.0, 4.6)**	1.5 (-0.5, 3.5)	-1.8 (-3.7, 0.0)	1.5 (-0.1, 3.2)	**3.1 (0.1, 6.2)**	1.9 (-0.9, 4.7)	2.3 (-1.3, 6.0)
**Travel**^**h**^								
Yes	NA	3.7 (-3.9, 11.4)	**5.5 (2.2, 8.7)**	NA	NA	NA	NA	NA
No	NA	Referent	Referent	NA	NA	NA	NA	NA

*CI* confidence interval; *NA* not applicable.

**Note:** Boldface indicates significance (p<0.05 comparing to reference group).

^a^Adults were considered at high-risk for hepatitis A virus (HAV) infection if they reported any one of the following risk factors: ever traveled outside of the United States to countries other than Europe, Japan, Australia, New Zealand or Canada, since 1995 or if they had been ever told by a doctor; or other health professional that they had any kind of chronic, or long-term liver condition.

^b^Adults were considered at high-risk for hepatitis B virus (HBV) infection if they reported any one of the following risk factors: ever traveled outside of the United States to countries other than Europe, Japan, Australia, New Zealand or Canada, since 1995; or if they had been ever told by a doctor or other health professional that they had any kind of chronic, or long-term liver condition; or if they had ever been told by a doctor or other health professional that they had diabetes.

^c^Adults were considered at increased risk for pneumococcal disease if they had ever been told by a doctor or other health professional that they had diabetes, emphysema, chronic obstructive pulmonary disease, coronary heart disease, angina, heart attack, or other heart condition; had a diagnosis of cancer during the previous 12 months (excluding nonmelanoma skin cancer); had ever been told by a doctor or other health professional that they had lymphoma, leukemia, or blood cancer; or they had been told by a doctor or other health professional that they had chronic bronchitis or weak or failing kidneys during the preceding 12 months or had an asthma episode or attack during the preceding 12 months; or they were current smokers.

^d^Adjusted prevalence differences, adjusted for age, sex, race/ethnicity, education, employment status, poverty status, physician contact within the past year, usual place of healthcare, health insurance status, self-reported smoking status, five or more alcoholic drinks in 1 day at least once in past year, HIV test status, self-reported health status, obesity status, asthma status, diabetes status, cardiovascular disease status, chronic liver disease status, disability status, met Federal guidelines for aerobic physical activity, and sexual orientation.

^e^Adjusted prevalence differences, adjusted for age, sex, race/ethnicity, education, employment status, poverty status, physician contact within the past year, usual place of healthcare, health insurance status, self-reported smoking status, five or more alcoholic drinks in 1 day at least once in past year, HIV test status, self-reported health status, obesity status, asthma status, diabetes status, cardiovascular disease status, chronic liver disease status, disability status, met Federal guidelines for aerobic physical activity, sexual orientation, and travel.

^f^Estimate is not reported because it is unreliable either due to effective small sample size (n<30) and/or CI half-width >15 as per the National Center for Health Statistics Data Presentation Standards for Proportions, Series 2 Report available at: https://www.cdc.gov/nchs/data/series/sr_02/sr02_175.pdf.

^g^Poverty status was defined according to the ratio of a family’s total income to the federal poverty threshold. Federal poverty thresholds are updated annually by the U.S. Census Bureau (https://www.census.gov/data/tables/time-series/demo/income-poverty/historical-poverty-thresholds.html).

^h^Persons from developed countries who travel to developing countries with high or intermediate HAV and HBV endemicity are considered at substantial risk for acquiring HAV and HBV infections. Persons who traveled outside the United States to countries other than Europe, Japan, Australia, New Zealand, or Canada were considered having traveled to countries with high or intermediate HAV and HBV endemicity.

## Discussion

Significant differences were observed by sexual orientation for HepA, HepB, and influenza vaccination. Gay/lesbian orientation was associated with a greater likelihood of influenza vaccination. Gay male sexual orientation was associated with greater likelihood of HepA and HepB vaccination.

This study found that HepB vaccination was higher among men who self-identified as gay compared with heterosexual, consistent with findings reported previously [29,46] but lower than reported from studies involving smaller samples of gay males from localized geographical areas and settings [47,48]. Gay/lesbian and bisexual adults also had a higher likelihood of receiving HepA and HepB vaccination. The HepA vaccination coverage observed among gay males (40.3%) in this study was lower than previously reported (69.0%) [48]. Despite recommendations for HepA and HepB vaccination of MSM due to their increased risk for HAV and HBV infection [3,5], many gay males and bisexual males have not been vaccinated. More gay men than bisexual men have been reported to disclose to their primary care providers about their sexual encounters with men [29], thus may provide healthcare providers more opportunity to recognize the need for and recommend HepA and HepB vaccination to gay men. This may help explain the observed higher HepA and HepB vaccination coverage in this population. Having self-identified as gay/lesbian orientation was associated with greater likelihood of influenza vaccination in our multivariable analysis, with bisexual adults having lower influenza coverage than gay/lesbian and heterosexual adults. That bisexual adults were less likely to have health insurance or a usual place for health care, and more likely to be below poverty in this study might be contributors to this finding.

Because HepA, HepB, and HPV vaccinations protect against sexually transmitted infections, persons at higher risk of acquiring these infections because of risky sexual behaviors (e.g., MSM) might be expected to have higher coverage for those vaccines. Though HepA and HepB vaccination coverage among gay males and bisexual females was significantly higher than heterosexual males, HepA and HepB vaccination coverage among high-risk adults remains suboptimal and most remain at risk for HAV and HBV infection. As new HBV infections continue to occur with cumulative exposure over time and transmission among MSM continues during adulthood [49], until the vaccinated young adults age, which over time may increase the vaccine-induced population immunity, “catch-up” HepA and HepB vaccination campaigns among the unvaccinated, at-risk young adult population [3,5] might help improve coverage. Additional strategies to improve HepA and HepB vaccination among adults at increased risk might include: encouraging healthcare providers to identify candidates for HepA and HepB vaccination and ensuring that all adults at risk for HAV and HBV infection or who seek protection from HAV and HBV infection are offered HepA and HepB vaccines [3,5,50]; healthcare providers providing environments that facilitate both gay and bisexual men disclosing their sexual behaviors and other risk factors, possibly by posting non-discrimination statements in their clinics, encouraging openness in patient-provider discussions, using gender-neutral language about sexual partners, and discussing sexual health issues openly using nonjudgmental questions about sexual practices and behaviors [51–53]; and routine provision of HepB vaccine possibly at settings serving gender minorities, such as HIV counseling and testing sites that are able to provide vaccination.

This study used multivariable logistic model(s) to identify factors associated with vaccination. For HepA and HepB vaccinations, the findings from the multivariable models were driven mainly by males. A significantly higher percentage of gay men had received HepA and/or HepB vaccinations than their heterosexual counterparts, possibly reflecting differential implementation of recommendations for vaccination of individuals at increased risk due to sexual behavior versus travel to countries in which HAV or HBV are endemic or having diabetes or chronic liver disease [2], differential recall of vaccination by the at risk population, as well as additional unmeasured confounding factor(s). A significantly higher percentage of gay/lesbian adults had received influenza vaccination than heterosexual adults, but not Tdap, pneumococcal or shingles vaccination. This observation is not well understood given the greater use of health services among gay/lesbian adults and the generally higher acceptability of vaccines in these groups [29]. In the multivariable models, however, no specific causal model or hypothesis was posed for the relationship between vaccination (for each specific vaccine) and the demographic, behavioral, health status, access to care characteristics, and sexual orientation considered in this report. Thus, although multiple factors were identified as having an independent association with a higher or lower likelihood of vaccination, the relative importance of these factors with respect to their association with vaccination cannot be determined from this analysis. The models were successful, however, in identifying factors generally associated with vaccination. Similar to other reports, characteristics associated with greater likelihood of vaccination included higher education, having health insurance, having had at least one or more physician contacts within the past year, and having a usual place to go for healthcare, even when controlling for sexual orientation [46,48].

Overall self-identified sexual orientation estimates from this report were similar to that reported from state-level and national surveys [17,18,54]. The estimates found in this report were lower compared with sexual orientation estimates reported elsewhere [55–57]. The differences in point estimates might be due to differences in sampling design, mode of the surveys, or other survey attributes. With the inclusion of questions on sexual orientation for the first time in the NHIS in 2013, it became possible to examine differences in self-reported receipt of selected vaccines by self-reported sexual orientation in a sample of U.S. adults from a national probability-based survey. Further, we also observed other important differences in respondent characteristics by sexual orientation, similar to those previously reported [1,2,15–19,31,52–54,58–60].

Several limitations should be considered in interpreting the results of this study. First, the information on vaccination was self-reported and may be subject to recall bias. However, adult self-reported vaccination status has been shown to be ≥70% sensitive in one or more studies for influenza, pneumococcal, tetanus toxoid-containing, herpes zoster, and HepB vaccines and ≥70% specific in one or more studies for all except tetanus and HepB vaccination [61–65]. Second, adult HepA and HepB vaccination are recommended for all unvaccinated adults at risk for HAV and HBV infection and for all adults requesting protection from HAV and HBV infection with the acknowledgment that a specific risk factor should not be a requirement for vaccination [3,5]. Because the NHIS does not collect information on all risk conditions for HAV and HBV infections, we were unable to identify all adults who were at increased risk for HAV and HBV infection and report vaccination estimates for these groups. Also, as the NHIS does not include measurement tools to assess whether or not prevention methods were used by the study sample while engaging in risky sexual behaviors, we could not conduct additional analysis for HepA and HepB vaccination by stratifying on adults self-identifying as gay/lesbian or bisexual that practiced sexual behaviors that put them at high risk for HAV and HBV infection. Third, the response rates for the three survey years in the report were 61.2%, 58.9%, and 55.2%. Nonresponse bias can result if respondents and nonrespondents differ in their vaccination rates and rates of other characteristics. Fourth, the differences observed in findings across studies could be a result of the differences in survey design, the mode of the surveys, question wording between NHIS and other data sources, and possible differential nonresponse by sexual orientation. Fifth, respondents with responses of “something else” (0.2%), “I don’t know the answer” (0.5%), and refused (0.5%) for the sexual orientation questions were classified as missing and excluded from the analyses. Although a small proportion of the overall sample, these exclusions create a small potential for bias and prevent assessment of the health indices and vaccination status of less populous groups (e.g., persons who identify as transgender). Sixth, the Tdap estimate is subject to considerable uncertainty. Respondents who reported a tetanus vaccination but were unable to say whether Td or Tdap was used during 2005–2015 were excluded from estimations of Tdap coverage (36.7%), creating a potential for bias. Seventh, despite combining three years of data for a larger sample size, the number of adults self-identifying as gay/lesbian, or bisexual was still relatively small when stratified across multiple covariate categories, which might cause some estimates to be unstable. The results pertaining to gay/lesbian or bisexual groups should be interpreted with caution. As additional years of data become available, more stable estimates could be generated. Eighth, the NHIS is a general population survey. The weighting is intended to represent the general population, not specifically populations defined by sexual orientation. Therefore, the estimates among gay, lesbian or bisexual respondents in this study might not be generalizable to the entire gay, lesbian or bisexual population [56,57]. Additionally, as noted, the sample size of persons in the NHIS who identified as gay, lesbian or bisexual is relatively small, which limited the ability to report coverage estimates by individual survey years. Ninth, as there are no unique personal identifiers in the NHIS that would allow determination of the proportion of respondents that might be survey repeaters across the years included in this study, it would be difficult to know if participants from each year were unique from other years. When the sociodemographic and health-related factors of the survey respondents from individual years (2013 vs. 2014, 2013 vs. 2015, 2014 vs. 2015 [data not shown]) were compared, similar patterns of similarity and differences were observed across the years, suggesting participants from each were unique from other years. Finally, though we controlled for potential confounders in this study, there might be unknown confounders that were not controlled for that might have biased the study estimates, the extent of which is hard to estimate.

Despite these limitations, study strengths include findings from a national probability-based survey that included noninstitutionalized U.S. adults who self-identified as gay/lesbian and bisexual that allowed national level vaccination coverage estimation by sexual orientation. This study is the first to assess associations of sexual orientation with a comprehensive list of vaccinations and could serve as a baseline for monitoring vaccination coverage changes over time.

## Conclusions

This study helps document differences in self-reported vaccination coverage by self-identified sexual orientation among U.S. adults aged 18 years and over and is the first to assess associations of sexual orientation with a comprehensive list of vaccinations. Findings from this study can serve as a baseline for monitoring vaccination coverage changes over time, and can assist the development of targeted strategies to improve the health status of lesbian, gay, and bisexual populations. Annual data on sexual orientation from the NHIS can be useful in monitoring the impact of policies and interventions directed at improving the health of those who identify as gay, lesbian and bisexual.
